# Differential responses of the intestine and liver transcriptome to high levels of plant proteins in diets for large yellow croaker (*Larimichthys crocea*)

**DOI:** 10.3389/fgene.2025.1540305

**Published:** 2025-03-05

**Authors:** Qiaozhen Ke, Yin Li, Huasong Weng, Baohua Chen, Jiaying Wang, Ji Zhao, Pengxin Jiang, Peng Xu, Tao Zhou

**Affiliations:** ^1^ College of the Environment and Ecology, Xiamen University, Xiamen, China; ^2^ State Key Laboratory of Mariculture Breeding, College of Ocean and Earth Sciences, Xiamen University, Xiamen, China; ^3^ State Key Laboratory of Mariculture Breeding, Ningde Fufa Fisheries Company Limited, Ningde, China

**Keywords:** Larimichthys crocea, carnivorous fish, high plant protein, transcriptome, nutrition metabolism

## Abstract

Large yellow croaker is an economically important carnivorous marine aquaculture fish in China with high protein requirements. Current fish meal - based feeds face issues like high cost and resource depletion, while plant protein sources have potential but also controversies. To explore this, a 120 - day feeding trial was conducted with a standard commercial feed (CF) and a modified feed (PF) where 70% of fish meal was replaced by plant protein. Results showed no significant growth performance differences between the two groups. Transcriptome analysis identified 557 and 308 differentially expressed genes in the liver and intestine respectively. GO and KEGG enrichment analyses indicated their association with immune response, lipid metabolism, and signal transduction. Five key genes related to metabolism and immune regulation were also found. These findings underscore the potential of integrating plant protein into fish diets, which could significantly enhance sustainable practices in global aquaculture while reducing reliance on fish meal. Emphasizing this transition is crucial for fostering environmental sustainability and supporting the future of aquaculture.

## Introduction

Large yellow croaker (*Larimichthys crocea*) is an important marine aquaculture species in China, with annual production of more than 280,997 tons in 2022 (China Fishery Statistical Yearbooks). As a carnivorous fish, the large yellow croaker has a relatively high demand for protein (He et al., 2017; Gu et al., 2019). Currently, chopped or minced trash fish is the major diet for large yellow croaker (Hardy, 2010; Zhang et al., 2016). However, issues such as its low feed utilization, unsafe quality, pollution of water quality and disease have resulted in a waste of resources and posed a great threat to the water environment and food safety (Shi et al., 2017; Kumar, 2019; Liu and Peng, 2021). The emergence of fish meal in feed formulations has effectively addressed these issues (Liu and Peng, 2021). However, its high cost has become the primary limiting factor for its use. The main ingredients of the complementary feed, such as fish meal and fish oil, heavily rely on imports. Nevertheless, global marine oil and fish meal resources are depleting gradually (Han et al., 2018). Additionally, the aquaculture industry is experiencing exponential growth to provide affordable protein for the world’s ever-increasing population, resulting in heightened feed demand. Large-scale overfishing for fish meal production has led to a decline in marine species (Zhou et al., 2015), and severely disrupted ecological balance. Fish oil (FO) and fish meal (FM) may further exacerbate the pressure on dwindling marine fish resources (Aladetohun, 2013; Oliva-Teles et al., 2022), and the shortage of fish meal has caused persistent price increases. In Asia alone, the consumption of fish meal for Nile tilapia rose from 800,000 tons to 1.7 million tons, while the production of fish feed increased from 40% in 2000 to 60% in 2008 (Tacon and Metian, 2008). According to the Food and Agriculture Organization of the United Nations, reducing the inclusion of fish meal and fish oil in feeds represents a significant advancement in alleviating the strain on global marine resources (Subasinghe, 2001). Therefore, with the expansion of aquaculture, major factors such as increased demand, uncertain supply, and high prices of fishmeal make it necessary to find alternative sources of fish meal (Mu et al., 2017).

Compared with animal protein sources, plant protein sources have the advantages of being widely available and inexpensive. Moreover, plant protein sources can protect marine fishery resources and promote sustainable development of marine fisheries (Shahin et al., 2023). In some feeds for practical applications for carnivorous fish, a reasonable mixture of plant protein ingredients can achieve a complete replacement of fishmeal without affecting their growth performance, such as *Sparus aurata* (Gómez-Requeni et al., 2004), *Rachycentron canadum* (Salze et al., 2010), *Oncorhynchus mykiss* (Lee et al., 2010) and *Dicentrarchus labrax* (Kaushik et al., 2004). However, compared to animal protein sources, the lack of certain essential amino acids (EAA) in plant protein components and the low utilization and palatability of it, have caused the controversy on the effectiveness of plant protein substitution (Dani, 2018), while the use of certain plant protein components or a high level substitution in fishmeal may slow down the growth of fish (Li et al., 2010; Espe et al., 2012). Therefore, the use of plant proteins in fishmeal should be more rigorous. When applying plant protein to large yellow croaker compound feeds, it is necessary to study the tolerance of large yellow croaker to plant protein, and experimental fish fed with different proportions of plant protein feeds are evaluated and molecular studies are performed.

Transcriptome analysis enables high-throughput screening of thousands of expressed genes in specific tissues (Damon et al., 2013). Through the identification of key genes and pathways, transcriptional regulatory mechanisms can be resolved, providing a theoretical reference and basis for the rational design of efficient large yellow croaker feed formulations (Li et al., 2007). Currently, numerous reports have explored the molecular mechanisms underlying the replacement of fishmeal with plant protein in various aquaculture species using transcriptome analysis. For example, In *Salmo salar*, Król et al. found that anti-nutritional factors (ANFs) present in plant proteins induce inflammatory responses in the intestines (Król et al., 2016). Caballero-Solares et al. reported that plant a protein-based diet leads to metabolic and immune changes in the liver (Caballero-Solares et al., 2018). *In Trachinotus ovatus*, Fan et al. observed that a high protein-based diet significantly affects immune and metabolic molecular changes in the liver (Fan et al., 2021). In *O. mykiss*, Panserat et al. demonstrated that a 100% plant-based diet induces significant metabolic changes in the liver (Panserat et al., 2009). Moreover, Cao et al. integrated transcriptomic and metabolomic analyses to show that plant protein feeding inhibits immune, metabolic, and protein functions (Cao et al., 2022). In large yellow croaker, research studies have shown that a diet with low plant protein replacing fishmeal does not significantly affect growth, immunity, or physiology (Li et al., 2010; Zhang et al., 2008; Wang et al., 2017). As a result, current commercial feed for large yellow croaker typically includes a low amount of plant protein. However, there have been no reports on the effects of high plant protein replacement for fishmeal or on transcriptome analysis.

Transcriptomic research allows us to understand the subtle effects of plant protein substitution for fish meal at the molecular level, enabling the selection of appropriate plant-based feed formulations to promote international sustainable development goals and maintain the healthy growth of the aquaculture industry. Therefore, in this study, we conducted a 120 days feeding experiment by using diet with standard commercial feed and a modified version, comparing the growth performance of large yellow croaker between different feed groups. We investigated differences in intestinal and liver transcript expression in experimental fish between feed groups to gain insight into how feed-induced differences in intestinal and liver transcript levels affect the overall physiology of the fish. This research aims to investigate the molecular-level changes in large yellow croaker resulting from plant protein substitution in the diet, focusing on its impact on metabolism and immunity, and providing a foundation for developing suitable aquaculture feed formulations.

## Materials and methods

### Experimental diets

We utilized four primary protein feed ingredients from standard commercial feed: fish meal, vital wheat gluten, dehulled soybean meal, and wheat flour, to adjust the protein composition and design the modified feed formula. Dehulled soybean meal and vital wheat gluten are inexpensive, readily available, and has a high protein content (Zlaugotne et al., 2023), while vital wheat gluten is a high-protein ingredient with an intriguing amino acid profile, particularly high in glutamine, which can improve gut health and modulate immunity (Apper-Bossard et al., 2013). The combination of these two ingredients effectively supplements the necessary amino acids and proteins.

The groups were designated as CF for the commercial feed group, which replaces 30% of the old formulation with plant protein. This formulation has been widely used in feeding large yellow croaker. The PF group, on the other hand, replaces 70% of the old formulation with plant protein, leading to a 37% substitution of plant protein compared to the CF group. The ingredient composition and main nutritional component for the CF and PF groups are shown in Table 1, with fish meal contents of 48% (CF) and 30% (PF). Fish meal, dehulled soybean meal, and vital wheat gluten were used as the main protein sources for the experimental feeds, while fish oil and soy lecithin were used as lipid sources. In the PF group, fish meal was reduced by 70% and replaced with plant protein. The ratio of dehulled soybean meal to vital wheat glutende was 1:3. To maintain equivalent protein and fat content in both feed groups, adjustments were made to the quantities of wheat flour and fish oil. The all experimental feeds were produced in Fujian Gaolong Co., Ltd., and made into puffed pellet feeds with a particle size of 4 mm.

**TABLE 1:** Ingredient content and proximate analysis of the experiment diets.

Group	Old formulation	CF	PF^a^
Ingredients (g/kg)			
Fishmeal	621.1	482.4	301.2
Vital wheat gluten	0	43	100
Dehulled soybean meal	0	135	315
Wheat flour	220	220	150
Fish oil	35	45.7	59.9
Soybean lecithin oil	15	15	15
Mineral premix	10	10	10
Vitamin premix	2	2	2
Vitamin C phosphate	1	1	1
Beer yeast	12.5	12.5	12.5
Beta glucan	0.9	0.9	0.9
Light calcium phosphate	20	20	20
Compound attractant	6	6	6
Preservatives	1	1	1
Antioxidants	0.5	0.5	0.5
Choline	5	5	5
Nutrient composition (% dry weight)			
Dry matter	93.59	92.84	91.38
Crude ash	12.66	12.40	9.72
Crude protein	47.38	46.09	45.90
Crude lipid	10.11	10.92	9.79

^a^ Specific adjustments were made to enhance plant-based protein inclusion by substituting a considerable amount of fishmeal with dehulled soybean meal and vital wheat gluten, thereby reducing reliance on fishmeal.

### Feeding management and sampling

The experimental large yellow croakers were obtained from the mariculture base of Ningde Fufa Fisheries Co., Ltd., (Ningde, Fujian, China), and the whole culture experiment was conducted in the culture sea area of Sandu’ao Dawan, Ningde. Fish from the same breeding batch, Similar sized large yellow croakers were temporarily reared in a large cage (8 m × 8 m × 5 m) to adapt to the experimental conditions, and were fed commercial feed for 2 weeks. Before the start of the different feed feeding culture experiments, the experimental fish were not fed for 24 h. These fish were randomly assigned to two experimental culture cages (8 m × 4 m × 5 m), with 2,000 fish per cage, and all the experimental cages were set up in the same marine environment. The cages were labeled as FC and PC groups in turn and fed with the corresponding labeled feeds. Each feed was fed once a day (17:00) until apparent satiation for 120 days. During the entire feeding experiment period (24 June 2019 to 22 October 2019), the seawater surface temperature was between 24.8°C and 28.6°C. The experimental fish were maintained under natural light conditions, meaning they were exposed solely to the available natural sunlight in the environment, without any artificial lighting. Consequently, the lighting conditions fluctuated in accordance with the natural day-night cycle.

Before the start of the experiment, 60 experimental fish were randomly picked and weighed after being anesthetized with eugenol (1:10,000) (Sinopharm Chemical Reagent Co., Ltd., SCR, Shanghai, China). After 120 days of feeding, 60 large yellow croakers were randomly selected from each group and weighed to determine the final body weight of the two groups of experimental fish to evaluate the growth performance of large yellow croaker. The parametric t-test was used to analyze the significant differences between groups in the initial body weight and final body weight of the experimental fish.

Compared to other organs, the intestine and liver are crucial for fish metabolism and nutrient absorption, and plant protein diets have a direct impact on their functions (Hussein et al., 2023; Dawood, 2021). Therefore, gene expression analysis of the intestine and liver is a primary focus of our study. Six fish (three for liver sampling and three for intestinal sampling) from each feed group were anesthetized with eugenol, then euthanized and sampled to minimize stress and suffering during the trial. During sampling, the abdominal cavity was cut open, and liver samples were immediately snap-frozen in liquid nitrogen and stored in an ultra-low temperature refrigerator at −80°C for subsequent RNA extraction. The intestinal tract was excised and the intestinal contents were removed. The intestinal segment under study was taken from the hindgut, which is defined as the region from the increase in intestinal diameter and presence of visible folds to the rectum, was subsequently cleaned of mesenteric and adipose tissue. Intestine samples were immediately snap-frozen in liquid nitrogen and stored in an ultra-low temperature refrigerator at −80°C for subsequent RNA extraction.

### RNA extraction, library construction and sequencing

Total RNA of the intestine and liver tissues was extracted using TransZol Up Plus RNA Kit following the manufacturer’s instructions. RNA purity was checked using the NanoPhotometer^®^ spectrophotometer (IMPLEN, CA, United States). RNA quantity and integrity was assessed using the RNA Nano 6000 Assay Kit of the Bioanalyzer 2100 system (Agilent Technologies, CA, United States). The RNA samples with OD260/280 values ranging from 1.67 to 2.16, OD260/230 values ranging from 1.23 to 2.80 and RNA Integrity Number (RIN) ≥ 6.5 were used for the subsequent library construction. A total of 18 RNA-Seq libraries were constructed using the TruSeq RNA Sample Preparation Kit (Illumina) and then sequenced on an Illumina HiSeq X Ten platform, which generates 150 bp pair-end reads.

### Identification of differentially expressed genes

After sequencing, the adaptor sequences and low-quality reads (quality score ≤ 20) were eliminated to obtain high quality clean reads using fastp (Chen et al., 2018). These clean reads were then aligned to a reference genome of *L. crocea* (NCBI accession: GCA_003845795.1) using HISAT2 (Kim et al., 2015). Cufflinks was used to assemble genes and identify differential expressed genes (DEGs) between control and treated group (Trapnell et al., 2013). In order to eliminate the influence of different gene lengths and sequencing discrepancies, we measured the gene expression level by the fragments per kilobase of exon model per million mapped reads (FPKM) based on the number of uniquely mapped reads. DESeq2 (Love et al., 2014) was used to analyze differential gene expression, and genes with statistically significant expression differences (*p-value* < 0.05, |log2(foldchange)| > 2) were considered to be DEGs.

### Functional annotation, GO and KEGG classification, protein-protein interaction analysis

All DEGs were functionally annotated based on the reference genome of *L. crocea*, which were annotated against the following four databases: NCBI non-redundant protein database (NR), Swiss-Prot database, Gene Ontology (GO) database and Kyoto Encyclopedia of Genes and Genomes (KEGG). In order to understand the function of these DEGs, GO functional enrichment analysis and KEGG pathway analysis were performed using the OmicShare tools (https://www.omicshare.com/tools/). The threshold for significant enrichment of gene sets was set at *p <* 0.05. Meanwhile, GO and KEGG enrichment analyses were performed on DEGs to explore their corresponding biological functions and associated pathways. The protein-protein interaction (PPI) network was constructed in STRING (https://string-db.org), a search tool for retrieving interacting proteins.

## Results

### Growth performance

**FIGURE 1:**
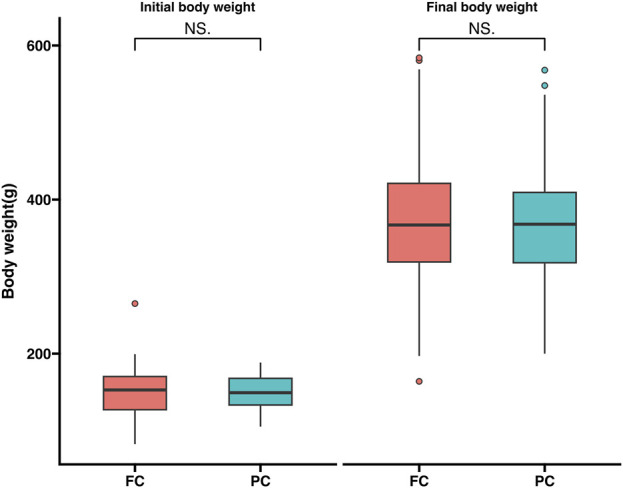
Comparison of growth performance among different levels of plant protein substitutes.

Before feeding the experimental feed 60 experimental fish were randomly picked and the initial average body weight was 150.8 ± 25.7 g. After feeding with the two experimental diets for 120 days, the final body weight of large yellow croaker in each experimental group were collected. The final weight of experimental fish in FC and PC were 372.4 ± 77.2 g and 367.0 ± 65.4 g, respectively (Figure 1). The results showed that there was no significant difference between the two groups (*p > 0.05*).

### Identification of DEGs between different feeding groups

Sequencing of 12 RNA samples from liver and intestine tissues yielded a total of 306,592,074 raw reads. After filtering, the number of clean reads of each sample ranged from 21,326,592 to 33,813,448. The clean reads rate of all samples was above 95% (Table 2). All clean reads were mapped to the large yellow croaker genome, and the average mapping rate of the 12 samples was 91.86%.

**TABLE 2:** Summary of RNA sequencing output and Clean reads mapping rate for liver and intestine samples.

Sample	Raw reads number	Clean reads number	Clean reads rate (%)	Mapping rate (%)
FC-L1	24,248,818	23,509,701	96.95	94.67
FC-L2	22,018,474	21,326,592	96.86	90.13
FC-L3	24,316,134	23,650,651	97.26	91.27
PC-L1	34,872,157	33,813,448	96.96	94.05
PC-L2	27,966,081	26,986,883	96.5	91.7
PC-L3	22,511,373	21,557,419	95.76	94.98
FC-I1	23,251,770	22,502,894	96.78	91.84
FC-I2	24,713,152	23,973,187	97.01	91.3
FC-I3	22,523,306	21,859,867	97.05	84.48
PC-I1	24,513,842	23,535,481	96.01	91.83
PC-I2	33,097,225	32,017,425	96.74	91.75
PC-I3	22,559,742	21,770,438	96.5	94.28

**FIGURE 2:**
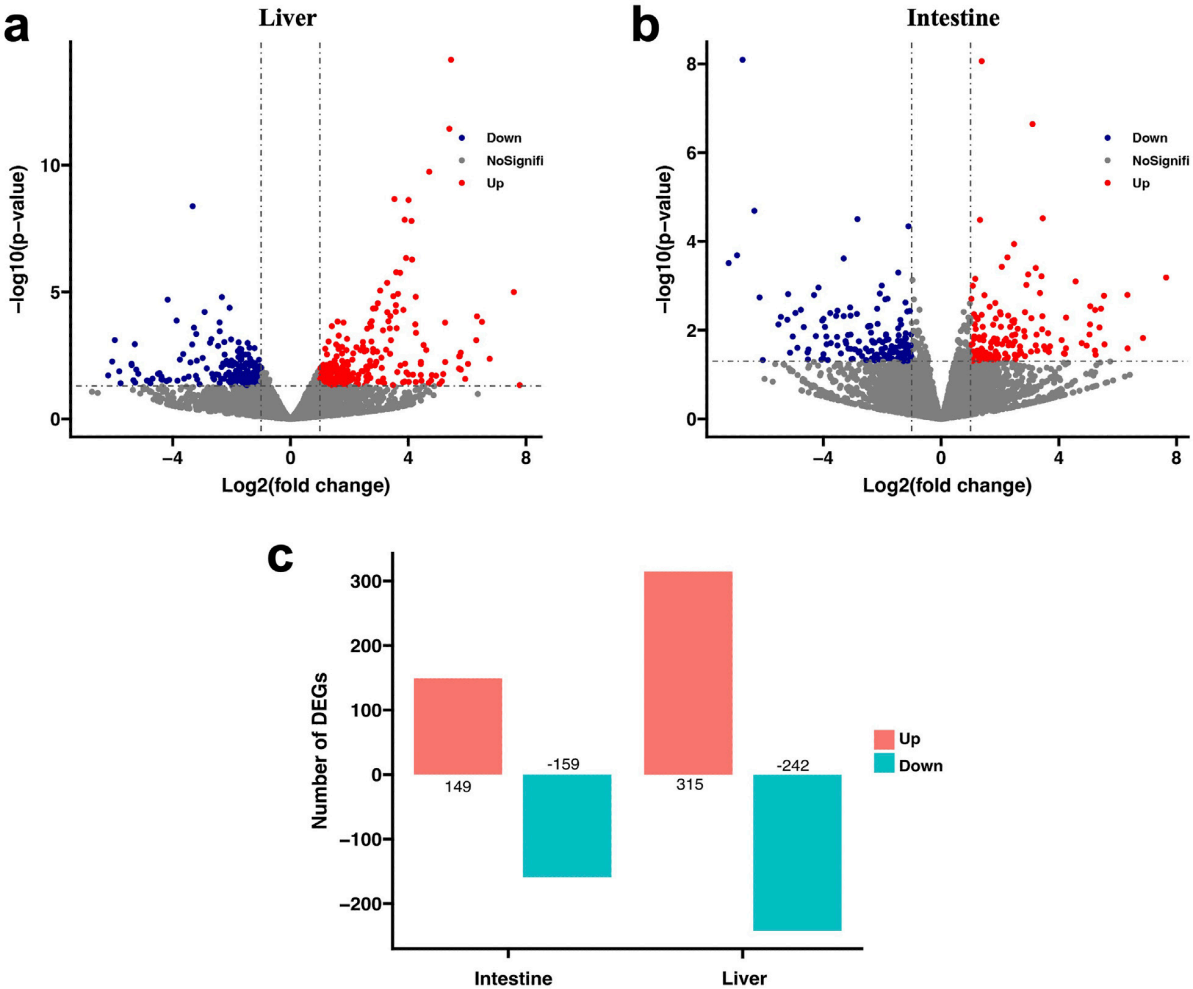
The volcano plot reveals the distribution of DEGs under different plant protein diets. **(A)** Volcano plot of DEGs in the liver. **(B)** Volcano plot of DEGs in the intestine. **(C)** Statistics of number of DEGs. Red spot: log_2_(fold change) ≥ 1, and *p <* 0.05; Blue spot: log_2_(fold change) ≤ −1, and *p <* 0.05; Black spot: no difference in expression.

By comparing the gene expression between the FC and PC groups, a total of 865 DEGs were identified. As shown in Figure 2, in the intestine tissue, the number of DEGs was 308, and in the liver tissue, it was 557.

### GO enrichment analysis of DEGs

**FIGURE 3:**
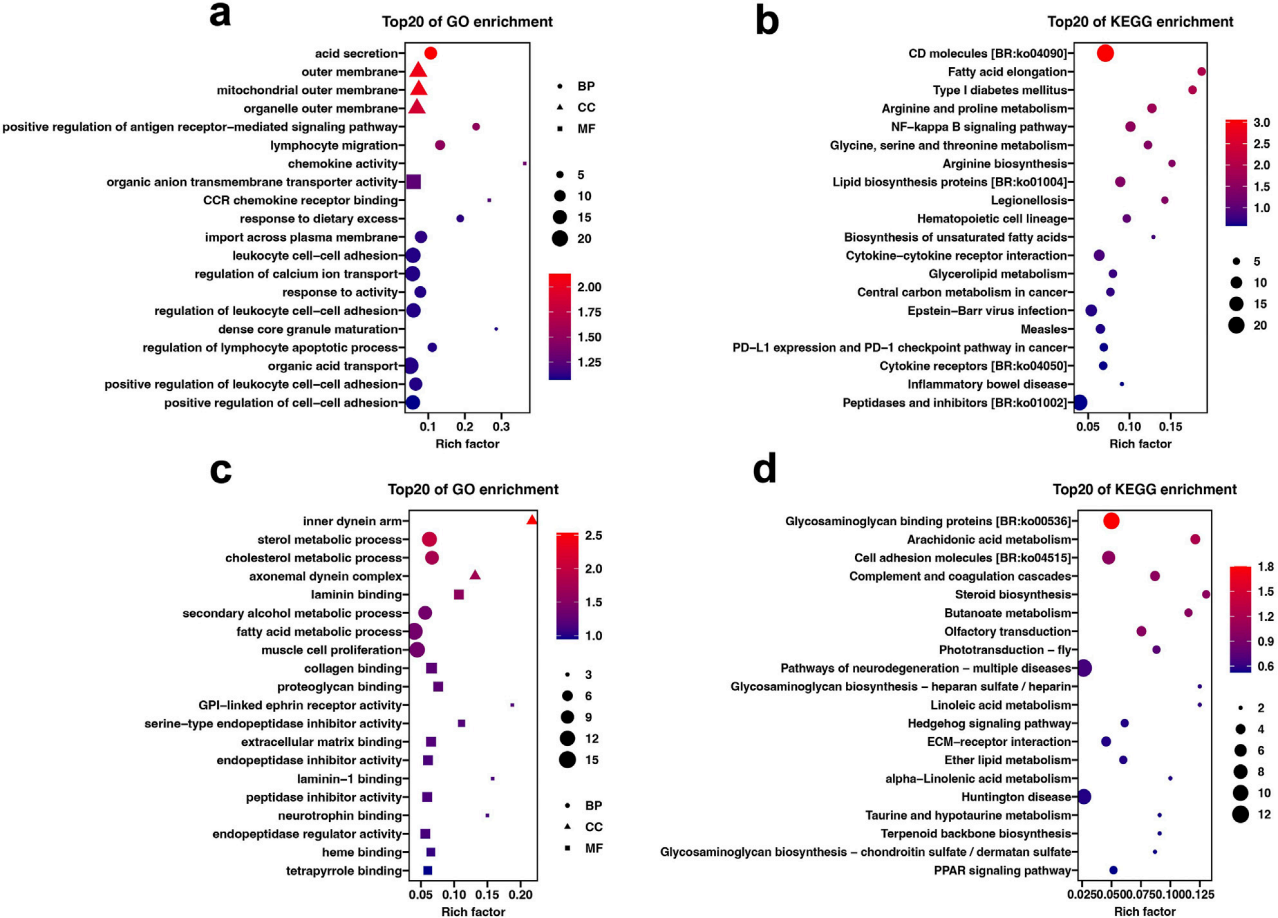
GO and KEGG enrichment analysis results of DEGs under different plant protein diets. **(A)** GO enrichment of DEGs in the Liver. **(B)** KEGG enrichment of DEGs in the Liver. **(C)** GO enrichment of DEGs in the intestine. **(D)** KEGG enrichment of DEGs in the intestine.

GO analysis showed that 557 DEGs in the liver tissue were classified into 671 GO terms (*p < 0.05*), including 538 in biological process category, 90 in molecular function categories, and 43 cellular component categories. GO terms related to immune and digestive absorption were significantly enriched, such as acid secretion, positive regulation of antigen receptor-mediated signaling pathway, and lymphocyte migration (Figure 3A; Supplementary Table 1). In the intestine tissue, 308 DEGs were classified into 378 GO terms (*p < 0.05*), including 292 in biological process category, 62 in molecular function category, and 24 cellular component categories. GO terms related to metabolism and intercellular interactions were significantly enriched, such as sterol metabolic process, fatty acid metabolic process, laminin binding, and collagen binding (Figure 3C; Supplementary Table 2).

### KEGG enrichment analysis of DEGs

KEGG analysis showed that 32 and 26 KEGG pathways were significantly enriched in the liver and intestine tissue (*p < 0.05*), respectively. In the liver tissue, pathways related to immune-related processes, such as CD molecules, NF-kappa B signaling pathway, and Cytokine-cytokine receptor interaction were significant enriched. In addition, metabolic pathways such as Fatty acid elongation, Glycine, serine and threonine metabolism, and Biosynthesis of unsaturated fatty acids were also significantly enriched (Figure 3B; Supplementary Table 1). In the intestine tissue, pathways related to cell signaling transduction, such as Glycosaminoglycan binding proteins, ECM-receptor interaction and Glycosaminoglycan biosynthesis-heparan sulfate/heparin were significantly enriched. Additionally, immune and metabolic pathways, including Arachidonic acid metabolism, Steroid biosynthesis and Butanoate metabolism, were also markedly enriched (Figure 3D; Supplementary Table 2).

### Regulation of immune regulation related gene expression

**FIGURE 4:**
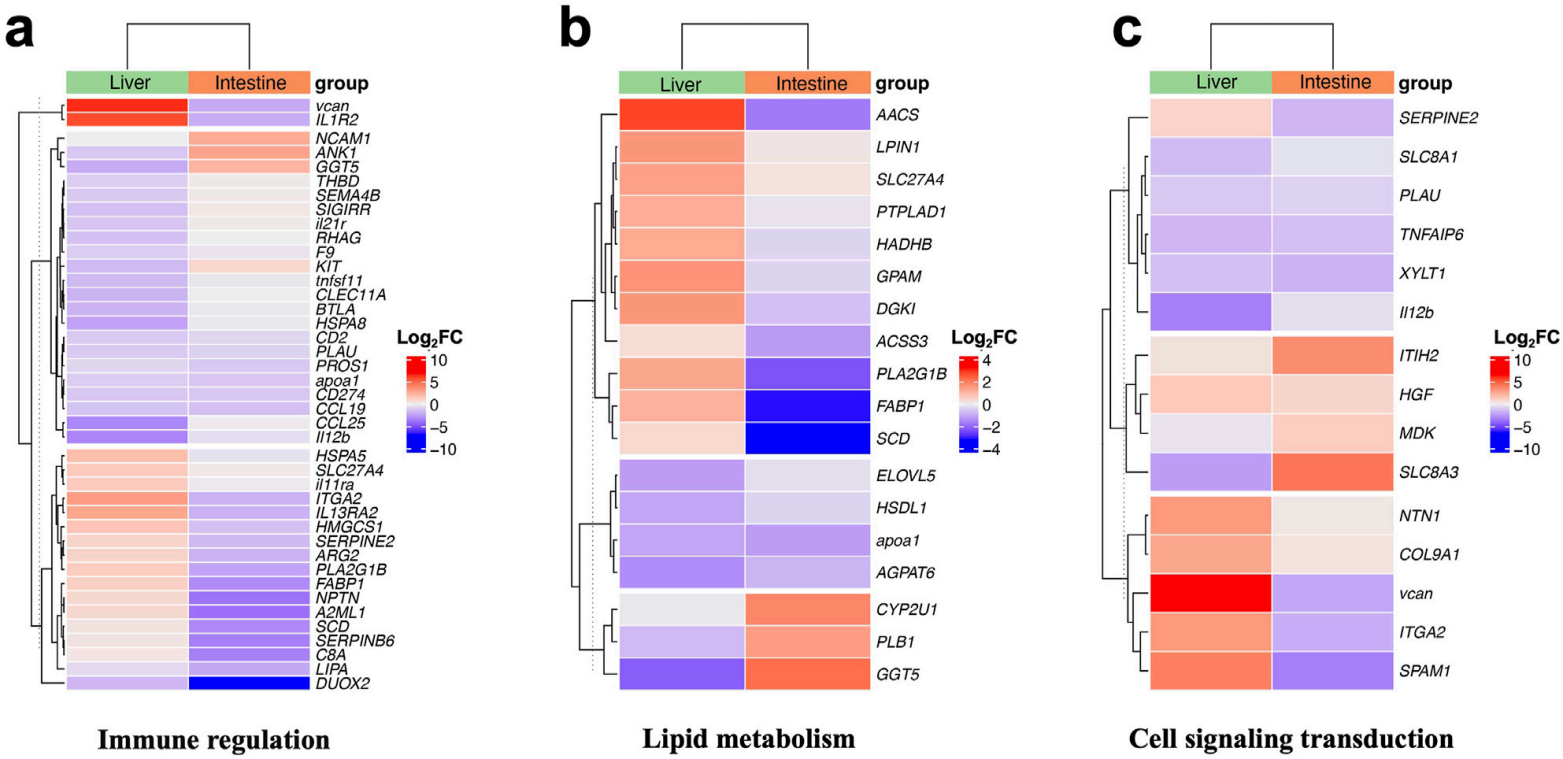
Heat map of DEGs expression (log2FC) in different pathways. **(A)** Immune regulation related gene expression. **(B)** Lipid metabolism related gene expression. **(C)** Cell singnaling transduction related gene expression.

To further elucidate the impact of high plant protein diet on large yellow croaker, we conducted additional statistical analysis on the KEGG results. In immune-related pathways, we identified a total of 41 genes. Expression analysis revealed a downregulation trend in immune-related genes after high plant protein feeding. In the liver group, 17 genes showed upregulation, while 23 genes exhibited downregulation. CCL25 and Il12b showed the most significant downregulation, while VCAN and IL1R2 showing upregulation (Figure 4A; Supplementary Table 3). In the intestine group, 9 genes showed upregulation, and 31 genes showed downregulation, with DUOX2 gene downregulation being the most pronounced.

### Regulation of lipid metabolism related gene expression

In lipid metabolism-related pathways, we identified a total of 18 DEGs. Expression analysis revealed that these genes were primarily upregulated in the liver. For instance, genes such as AACS, LPIN1, GPAM, and DGKI showed upregulation, with only a few genes like GGT5 showing downregulation (Figure 4B; Supplementary Table 4). In contrast, in the intestine, most genes such as AACS, FABP1, and SCD showed significant downregulation, with only a small number of genes like CYP2U1 and GGT5 showing upregulation.

### Regulation of cell signaling transduction related gene expression

In addition to the significant enrichment of pathways related to immune response and lipid metabolism, our enrichment analysis also revealed a considerable enrichment of genes associated with cell signaling transduction. We identified a total of 15 DEGs, and expression analysis showed that in the liver, 8 genes were upregulated while 7 genes were downregulated. Among them, the upregulation of the VCAN gene was most pronounced, while ll12b showed significant downregulation (Figure 4C; Supplementary Table 5). In the intestine, only 6 genes were upregulated, with SLC8A3 and ITIH2 showing the most notable upregulation, while among the downregulated genes, SPAM1 and VCAN exhibited the most significant downregulation.

### Protein-protein interaction network analysis

**FIGURE 5:**
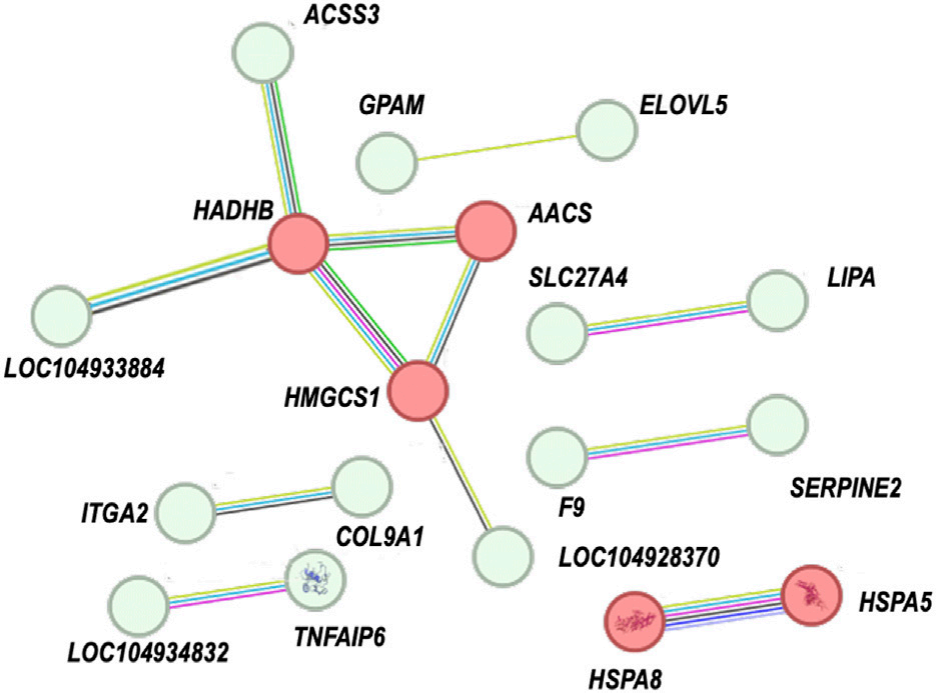
PPI network analysis of DEGs. The thickness of the line indicates the strength of the interaction between any two proteins, red colours indicate interaction scores greater than 0.7.

Furthermore, we performed a protein-protein interaction (PPI) network analysis on the DEGs related to immune response, lipid metabolism, and cell signaling transduction. As shown in the Figure 5 and Supplementary Table 6, numerous genes exhibit protein-protein interactions. Among them, five protein-coding genes—HADHB, HMGCS1, AACS, HSPA8, and HSPA5—had the highest levels of connectivity, with interaction scores greater than 0.7.

## Discussion

We compared the effects of standard commercial feed and a modified version, where 70% of the fish meal was replaced with plant protein, on large yellow croaker. We found that under the high plant protein feeding, the fish reached a growth rate similar to that of commercial feed feeding, indicating the feasibility of the high plant protein substitution strategy employed in this research. To deepen our understanding of the effects of plant protein substitution for fishmeal on fish physiology, we conducted an RNA-seq study to investigate the transcriptomic response of large yellow croaker under different plant protein replacement conditions. Extensive DEGs were observed through comparisons among different fishmeal replacement groups. Functional enrichment analysis of these DEGs revealed their association with various pathways, including immune regulation, lipid metabolism, and cell signaling transduction pathways, among others. These biological regulatory functions and processes may enhance our understanding of the response of fish to the substitution of fish meal with plant protein.

### Effect of plant protein diets on immunity regulation

Both GO and KEGG analyses revealed that many DEGs are involved in immune regulation pathways and functions, such as the positive regulation of antigen receptor-mediated signaling pathways, CD molecules, and the NF-kappa B signaling pathway. Notably, these pathways exhibited particularly pronounced enrichment in the liver, that may be consistent with the liver’s role as a central hub in immune regulation (Hussein et al., 2023). We observed that many genes were significantly upregulated in the liver, particularly VCAN and IL1R2. VCAN, an extracellular matrix protein, plays a critical role in tissue repair and inflammatory responses (Xu et al., 2021). Its chondroitin sulfate domain can directly bind to CD44, amplifying antigen presentation signals and promoting the production of inflammatory cytokines (Wight et al., 2014). IL1R2, as a key regulatory factor in the IL-1 signaling pathway, may inhibit the excessive activation of the downstream NF-κB pathway by competitively binding to the IL-1β ligand, especially when its expression level is elevated (Zhou et al., 2020; Zhang et al., 2020). Together, they are involved in inflammatory pathways such as CD molecules. The changes in the expression of these genes indicate that a plant-based protein diet has induced an inflammatory response in fish. This is also consistent with previous studies. For instance, Geay et al. found that plant protein diet significantly changed the immune levels in the liver of *Dicentrarchus labra* through immune indicators (Geay et al., 2011). Novriadi et al. reported that plant protein diet promoted inflammation levels in the liver of *Trachinotus carolinus* based on serum biochemistry analysis (Novriadi et al., 2018). In transcriptomic studies, changes in the expression of immune genes were also observed in the liver tissues of *O. mykiss* (Panserat et al., 2009) and *S. salar* (Caballero-Solares et al., 2018) following a plant protein diet. However, further analysis combining immune, biochemical, and morphological indicators is necessary to determine whether a plant protein diet would cause immune damage in large yellow croaker.

There are not many significantly enriched immune pathways observed in the intestine, and the expression of several key genes, such as DUOX2 and NPTN, is highly downregulated. DUOX2, as a core enzyme for the production of reactive oxygen species, when downregulated, may directly lead to a decline in the intestinal antibacterial capacity (Dobrzyn and Ntambi, 2005; Grasberger et al., 2015). NPTN, as a synaptic regulatory protein, potentially affecting the signaling between the enteric nervous system and immune cells, leading to an imbalance in immune tolerance (Beesley et al., 2014). The significant downregulation of DUOX2 and NPTN suggests that the mucosal barrier function may be compromised. The intestine not only serves as a site for nutrient absorption but also plays a crucial role in immune function. Intestinal cells closely interact with immune cells such as macrophages, participating in antigen presentation and the regulation of T cell responses (Hershberg and Mayer, 2000). The downregulation of the aforementioned genes may indicate an immune imbalance in the intestine caused by a plant protein diet. Further observations will be conducted in conjunction with immune and immunological indicators. Understanding these mechanisms could provide insights into practical dietary formulations that optimize fish health and immune function amidst the growing reliance on plant-based feeds.

### Effect of plant protein diets on lipid metabolism

Differences in fatty acid composition between plant proteins and fish meal will inevitably affect the efficiency of lipid metabolism when fed over the long term (Dani, 2018). In this study, we identified DEGs related to fatty acid synthesis, transport, metabolism, and lipid synthesis and regulation, with most of them showing downregulation in the intestine. In the intestine, there are significantly more pathways and genes related to lipid metabolism compared to immune regulation. This may be aligned with the intestine’s primary function of nutrient absorption (Dawood, 2021). For example, the FABP1 gene, which is a key carrier involved in intracellular lipid transport, shows significant downregulation that may impair the absorption and intracellular distribution of long-chain fatty acids (Assaily et al., 2011). Additionally, the suppression of SCD expression will directly lead to a reduction in the synthesis of monounsaturated fatty acids (Li et al., 2019). The Acetoacetyl-CoA synthase (AACS) gene is also involved, and defects in AACS function may hinder lipid storage by inhibiting the conversion of ketone bodies to acetyl-CoA (Ohgami et al., 2003). Given these roles, we hypothesize that replacing fish meal with plant protein may result in inadequate absorption of the materials necessary for lipid synthesis in the intestine. Previous transcriptomic studies on other carnivorous fish have yielded similar results. For example, studies on *S. salar* (Król et al., 2016), *Seriola lalandi* (Dam et al., 2020) and *O. mykiss* (Lazzarotto et al., 2018) have shown comparable findings. Estruch et al. also found through proteomic analysis that the intestinal mucosal proteome of *S. aurata*, L. was disrupted following the feeding of plant protein fishmea (Estruch et al., 2020). Evidently, it can still cause damage to the intestine at a microscopic level.

It is noteworthy that the AACS gene is significantly upregulated in the liver. Fatty acids serve as another crucial precursor in cholesterol synthesis. Consequently, Acetyl-CoA participates in cholesterol synthesis through a series of biochemical reactions (Duan et al., 2022). Cholesterol is exclusively sourced from animal organisms, when plant components replace a significant portion of fishmeal, the dietary cholesterol levels in the feed will decrease substantially, which may lead to alterations in cholesterol regulation (Deng et al., 2009). For instance, in rainbow trout, coping mechanisms against changes in nutritional characteristics and the lack of dietary cholesterol supply involve increasing cholesterol synthesis and limiting cholesterol efflux (Zhu et al., 2018). The liver is the primary center for synthesizing and regulating cholesterol (van der Wulp et al., 2013). Therefore, we speculate that the elevation of cholesterol synthesis-related genes in the liver may be due to the lack of cholesterol supply from plant protein, prompting large yellow croakers to initiate their own biosynthetic mechanisms to produce cholesterol to meet their nutritional needs. This also demonstrates a critical interplay between these organs in maintaining metabolic homeostasis.

### Effect of plant protein diets on cell signal transduction

In our study, we identified numerous pathways and functional genes associated with cell signaling transduction, specifically the Glycosaminoglycan (GAG) binding proteins pathway, ECM-receptor interaction, and the PPAR signaling pathway. Glycosaminoglycans (GAGs) are integral components of the extracellular matrix (ECM) that significantly influence cell adhesion and signal transduction, functioning in the regulation of cell growth and immune modulation (Gandhi and Mancera, 2008; Zhang and Zhang, 2010). The ECM not only maintains tissue structure but also plays a critical role in lipid metabolism by creating a conducive microenvironment for lipid synthesis. Prior research, particularly in chickens, has demonstrated that ECM components can substantially impact lipid metabolism (Mariman and Wang, 2010), underscoring the interconnectedness between the ECM and lipid regulatory mechanisms. The PPAR signaling pathway is particularly notable for its role in coordinating lipid metabolism and inflammatory responses (Zhang and Young, 2002). PPARs (Peroxisome Proliferator-Activated Receptors) are nuclear receptors that regulate genes involved in fatty acid uptake, storage, and β-oxidation, linking lipid homeostasis with inflammation modulation. The interplay among these pathways is crucial. For example, GAGs may alter ECM properties, thereby influencing PPAR activation. Hence, changes in GAGs or ECM components could directly impact PPAR signaling, affecting both lipid metabolism and inflammatory processes. Although there are no previous studies addressing these pathways, their intrinsic functional relationships suggest that further investigation is warranted.

Moreover, we observed that the DEGs involved in signal transduction were predominantly downregulated, particularly in the intestine. For instance, VCAN, previously linked to immune pathways, is essential for tissue repair and inflammatory responses (Xu et al., 2021). ITGA2, an integrin family member, is critical to the innate immune response and engages in various cellular processes, including cell development and metabolism (Zhu et al., 2013; Takada et al., 2007). Evidence suggests its involvement in cold stress responses in *Oreochromis niloticus* (El-Sayed et al., 2023) and immune cell migration in *S. aurata* (Espinosa-Ruíz and Esteban, 2023). The downregulation of the VCAN and ITGA2 genes may be closely related to the expression of immune genes in the intestine, as these genes are likely involved in regulating the expression of immune-related genes. In contrast, the upregulation of these genes in the liver suggests a tissue-specific adaptation to fulfill the metabolic and functional demands of this organ. This stark contrast highlights that while the intestine experiences disruptions in immune and metabolic pathways due to the downregulation of signal transduction genes, the liver compensates by enhancing the expression of genes associated with protective and reparative roles. Understanding the relationships among these genes is critical as we explore the potential of plant protein sources to replace fish meal in aquaculture diets. By optimizing the processes influenced by these genes, we can enhance the nutritional profiles of plant-based diets, thereby supporting fish growth, immunity, and resilience.

### Protein-protein interaction network

Protein-protein interaction (PPI) networks are complex network structures formed by protein interactions. These networks reflect the interactions between proteins in an organism and help us better understand the complexity and regulatory mechanisms of biological systems. In this study, we identified PPI among some genes, and these genes are involved in the immune, metabolic, and cell signaling pathways we mentioned earlier. This indicates that high plant protein feeding leads to a series of integrated responses.

Among them, HADHB, HMGCS1, AACS, HSPA8, and HSPA5 exhibit the highest level of connectivity. HADHB, HMGCS1, and AACS are involved in regulating fatty acid metabolism (Ohgami et al., 2003; Xie et al., 2023; Gu et al., 2024). HSPA8, HSPA5, and others may play a role in immune responses (van der Wulp et al., 2013; Tian et al., 2023). Further research is needed to explore the relationships between their interactions. In conclusion, the discovery of these genes and their related functions further enhances the understanding of the mechanism behind high plant protein substitution for fish meal in feeding large yellow croaker. The interconnectedness of these genes highlights a complex regulatory network that mediates both metabolic and immune functions, illustrating how these processes are vital for adapting to high levels of plant protein substitution in diets. Further research is needed to explore the relationships between their interactions to fully understand how they collectively influence fish physiology. In conclusion, the discovery of these genes and their associated functions significantly enriches our understanding of the mechanisms underpinning the effective substitution of fish meal with plant protein in large yellow croaker diets.

### Preliminary exploration in plant protein utilization

For carnivorous fish such as the large yellow croaker, with the expansion of aquaculture production, feeding fresh mixed fish and high-fishmeal pellet feed not only leads to environmental pollution but also results in a shortage of fishmeal supply and high prices (Mu et al., 2017). However, achieving complete substitution with plant protein diet still poses challenges. It is necessary to have a deeper understanding of the molecular changes caused by plant protein diet. Research on plant protein diets has been conducted in various carnivorous fish species. For instance, in *S. salar*, replacing 45% of fishmeal with soy protein concentrate does not lead to significant differences in growth or intestinal inflammation. Similarly, in *S. lalandi*, replacing 50% of fishmeal with soy protein concentrate also does not result in notable differences in growth or intestinal inflammation (Król et al., 2016; Dam et al., 2020). In a high-plant protein fishmeal diet, replacing 60% of fishmeal with defatted cottonseed meal (CSM) and corn protein concentrate in *T. ovatus*, as well as substituting 80% of fishmeal with soy protein concentrate in *S. salar*, has been shown to promote inflammation (Caballero-Solares et al., 2018; Fan et al., 2021). Furthermore, high-plant protein diets have been associated with reduced growth rates. Similarly, in *O. mykiss*, a study by Panserat et al. that replaced 100% of fishmeal with corn protein concentrate and soybean meal over 9.5-week trial found a decrease in growth rates (Panserat et al., 2009). Additionally, Cao et al. conducted a continuous 84-day experiment using 80% soybean meal as a fishmeal substitute and also observed a decline in growth performance (Cao et al., 2022). In our study, we further modified the commercial feed to achieve a higher plant protein content. We found that the body weight changes in large yellow croaker caused by high plant protein diet are comparable to those with low plant protein feeding. This differs from the results observed in *O. mykiss* and *S. salar*, suggesting potential species-specific responses. Additionally, the large yellow croaker is currently commonly fed diets with 30% plant protein fishmeal, which may accelerate the adaptation to plant protein diets. However, from the perspective of transcriptome analysis, we also observed changes in immune and metabolic pathways influenced by plant protein. This suggests that a high plant protein diet may have potential effects, such as promoting inflammation, and the long-term effects still require further investigation.

Dehulled soybean meal and vital wheat gluten contain various anti-nutritional factors (ANFs), such as phytic acid, tannins, protease inhibitors, and oligosaccharides, which can affect growth, digestion, absorption, and antioxidant capacity, as well as cause damage to the intestines of aquatic animals (Lin and Cheng, 2017). In this study, we simultaneously analyzed molecular changes in both the liver and intestine and found that although the impact of a high plant protein diet on growth was minimal, there were observable effects at the molecular level on the immune function of the liver and metabolic processes in the intestine of large yellow croaker. We speculate that plant proteins may directly disrupt intestinal absorption and affect the immune function of the liver. While no differences were noted in the short-term feeding trials, the potential for long-term effects cannot be ruled out. Of course, our findings are specific to carnivorous fish, as herbivorous and omnivorous fish have a lower reliance on fishmeal compared to carnivorous species (Dhar et al., 2024). These findings offer significant insights into feed formulation, suggesting that it is crucial to acknowledge the potential trade-offs associated with high plant protein diets. To address the ANFs present in plant proteins, we recommend several actionable solutions. a) processing techniques such as heat treatment, fermentation, and enzymatic hydrolysis can be employed to reduce the levels of these factors, thereby improving nutrient availability (Handa et al., 2017; Mugwanya et al., 2023). b) Additionally, incorporating functional additives like inosine (Hossain et al., 2018) and taurine (Ye et al., 2019) into plant protein-based feeds can significantly enhance the availability of essential amino acids, improving health and disease resistance while mitigating the overall antioxidant effects of plant proteins. c) we suggest that aquaculture enterprises adopt a phased implementation framework, starting with small-scale trials at farming sites to evaluate effectiveness. Based on the results, the application can be gradually expanded, with regular monitoring of fish immune function and metabolic health to make timely adjustments to feed formulations. Continuous monitoring is essential to ensure the health and performance of the fish.

Our future research will focus on investigating the specific impacts of high plant protein diets on the immune system and metabolism of large yellow croaker. We will also explore ways to integrate immune-boosting ingredients, into feed formulations. These efforts aim to optimize feed while addressing concerns related to ANFs. Ultimately, these strategies not only promote improved fish health and growth but also contribute to more sustainable aquaculture practices.

## Conclusion

In this study, the response of transcriptome to high levels of fishmeal replacement was examined in large yellow croaker. Based on our research findings, a high plant protein diet for large yellow croaker achieved the same weight gain as a low plant protein diet. Transcriptome analysis results show that 557 and 308 DEGs in the intestine and liver were identified, respectively. GO and KEGG analyses indicated significant enrichment in metabolism-related pathways such as lipid metabolism, immune regulation, and cell signaling transduction pathways. Expression analysis revealed tissue-specific expression of these genes, and the PPI network further revealed their interactions, collectively participating in the molecular regulation of high plant protein diets. These insights are vital for developing optimized aquafeed that enhances sustainability in aquaculture. Future steps should include testing specific feed additives to mitigate immune suppression and extending this analysis to other fish species, particularly carnivorous fish, to enhance the adaptability of various species to high plant protein diets. This work contributes to the advancement of sustainable practices in the aquaculture industry.

## Data Availability

The datasets presented in this study can be found in online repositories. The names of the repository/repositories and accession number(s) can be found below: https://www.ncbi.nlm.nih.gov/, PRJNA1119058.

## Supplementary material

SUPPLEMENTARY TABLE 1 Enrichment results of differentially expressed genes in the liver.

SUPPLEMENTARY TABLE 2 Enrichment results of differentially expressed genes in the intestine.

SUPPLEMENTARY TABLE 3 Statistics of immune regulation related gene expression.

SUPPLEMENTARY TABLE 4 Statistics of lipid metabolism related gene expression.

SUPPLEMENTARY TABLE 5 Statistics of cell signaling transduction related gene expression.

SUPPLEMENTARY TABLE 6 Statistical results of the protein-protein interaction network.
